# The box office prediction model based on the optimized XGBoost algorithm in the context of film marketing and distribution

**DOI:** 10.1371/journal.pone.0309227

**Published:** 2024-10-03

**Authors:** Shenglan Tang

**Affiliations:** Kyonggi University, Suwon City, South Korea; Vellore Institute of Technology, INDIA

## Abstract

To improve the accuracy and efficiency of box office prediction, this study deeply discusses the application of the optimized eXtreme Gradient Boosting (XGBoost) model in this scenario and its advantages compared with other commonly used machine learning models. By comparing and analyzing five models, involving the Deep Neural Network, Light Gradient Boosting Machine, Random Forest, Gradient Boosting Decision Tree, and CatBoost, several key performance indicators such as accuracy, precision, recall, F1 score, generalization error, stability, robustness, and adaptability score are comprehensively investigated. The research results reveal that the optimization model proposed in this study is superior to the comparison model in most evaluation indicators, especially when the data volume reaches 2500, showing obvious advantages. For example, the accuracy is increased to 0.9, the F1 score is 0.9, the generalization error is reduced to 0.09, and the stability score is as high as 0.98. The robustness and adaptability scores are both 0.97, which proves its strong prediction ability and high stability and robustness on large-scale datasets. Therefore, this study provides scientific data support and a decision-making basis for the film industry in the formulation of marketing and distribution strategies. Moreover, film producers and distributors can reasonably estimate their market performance early in film shooting, optimize investment decisions, and reduce economic risks through accurate box office predictions.

## 1. Introduction

The film industry, as one of the most influential and economically valuable entertainment sectors globally, primarily manifests its economic benefits through box office revenue. With the advent of the digital era, revolutionary changes have occurred in film marketing and distribution strategies, transitioning from traditional methods such as posters and trailers to precise marketing through the use of social media and online platforms [1]. These changes have significantly increased the number and complexity of variables influencing box office performance, making accurate box office predictions more crucial. Precise box office predictions not only assist film producers and distributors in formulating more effective marketing strategies but also optimize resource allocation and reduce economic risks [2–4]. Therefore, developing an efficient and accurate box office prediction model holds vital practical importance for the film industry. In recent years, machine learning (ML) technology has found widespread application in the field of box office prediction, with the eXtreme Gradient Boosting (XGBoost) algorithm being widely recognized as a powerful tool for addressing such prediction challenges due to its efficiency, accuracy, and interpretability [5].

Research on the application of advanced ML models such as XGBoost, CatBoost, and RF has provided valuable insights, which are crucially inspiring for the research here. CatBoost, as a novel gradient boosting algorithm, excels particularly in handling classification problems and possesses unique advantages in handling missing values and categorical features. Luo et al. (2021) proposed a method for estimating aboveground biomass (AGB) using a classification boosting algorithm and compared different combinations of feature selection methods and ML algorithms [6]. The results of this study indicated that using a single ML algorithm for feature selection and regression may not always guarantee the best AGB estimation results. Therefore, applying new ML algorithms and feature selection methods for improving the accuracy of AGB estimation holds promising prospects. Dai et al. (2023) proposed a high-dimensional multi-objective optimal scheduling strategy for pollutant emissions from thermal power plants, considering the spatiotemporal distribution of various pollutants [7]. The study showed that this scheduling method not only effectively improved air quality but also adjusted according to the spatiotemporal variations of environmental capacity, thus achieving economic and environmentally friendly power scheduling. Dai and Wang et al. (2023) introduced a VAR-XGBoost model based on Vector Autoregression (VAR), the Kriging method, and XGBoost (Extreme Gradient Boosting) [8]. Their research concluded that ozone had the highest correlation with PM2.5 and the lowest correlation with sulfur dioxide. Among the measured factors, wind speed and temperature were among the most important factors influencing ozone concentration. By integrating the findings of these existing studies, a more comprehensive understanding of the advantages and limitations of XGBoost, CatBoost, and RF models can be achieved, thereby providing more solid theoretical support for the box office prediction model in this paper. Additionally, these studies inspire considerations regarding strategies for handling spatiotemporal data, missing values, and categorical features, and how to improve the robustness and accuracy of the model through ensemble learning methods. In future work, this study plans to delve deeper into the principles and applications of these algorithms, explore their potential in box office prediction, and attempt to integrate their advantages into the model proposed here. Furthermore, this study also focuses on the strategies employed by these models in handling similar complex problems, such as spatiotemporal data analysis, missing value handling, and optimization of categorical features, to further enhance the performance and generalization ability of the model proposed.

Although the existing XGBoost base model has shown excellent performance in several areas, the specificity of box office prediction, such as the diversity of film features, the uncertainty of marketing campaigns, and the complexity of consumer behavior, all require the model to be optimized to suit this particular area. In addition, with the progress of big data technology, the film industry has accumulated a large number of data resources, such as user ratings, movie reviews, social media emotional tendencies, etc. The effective use of these data is crucial to improve the accuracy of box office prediction.

This study proposes an optimized XGBoost algorithm-based box office prediction model, which demonstrates significant innovation in the field of film marketing and distribution. The following are the key innovations of this study:

1. Algorithm Optimization: The study meticulously optimizes the XGBoost algorithm. By adjusting parameters such as learning rate, maximum tree depth, subsample ratio, and column sample ratio, it significantly improves the model’s prediction accuracy and generalization ability. This optimization method is particularly effective in handling large-scale datasets, better controlling model complexity, and avoiding overfitting.
2. Integration of Multidimensional Features: The model of this study considers not only traditional movie metadata such as director, cast, and budget but also integrates new data sources such as social media sentiment trends and user reviews, thereby comprehensively capturing the multidimensional factors influencing box office performance.
3. Time Series Analysis: This study introduces time series analysis and dynamic feature adjustment mechanisms, enabling the model to adapt to changes in the market and audience interests, thereby improving prediction timeliness and accuracy.
4. Practical Application Orientation: The research not only focuses on the theoretical performance of the model but also emphasizes its decision support role in the actual film industry. By providing accurate box office predictions, it helps filmmakers and distributors optimize investment decisions and reduce economic risks.
5. Interdisciplinary Methodology: The research methodology of this study combines knowledge from ML, data science, and film marketing, offering a novel interdisciplinary solution for box office prediction.

These innovations not only advance the development of box office prediction technology but also provide the film industry with a new tool for formulating market strategies and allocating resources more scientifically.

## 2. Literature review

In previous research, Liao and Huang (2021) identified that box office revenue played a decisive role in assessing the commercial success of films. Accurate box office predictions could markedly enhance the film industry’s economic benefits and resource allocation efficiency [9]. He and Hu (2021) emphasized the crucial role of box office prediction in formulating effective film marketing and distribution strategies, providing film producers and distributors with a competitive advantage in a fierce market [10]. Qiu and Zheng (2023) confirmed through their study that using ML models for box office prediction outperformed traditional statistical methods in terms of accuracy and reliability [11]. Mbunge et al. (2022) demonstrated that ML models, combining film metadata and social media metrics, could effectively improve the accuracy of box office predictions [12]. Lu et al. (2022) explored methods for optimizing the performance of the XGBoost algorithm through parameter tuning and feature selection. They found that these optimization techniques notably enhanced the model’s predictive performance [13]. Knudsen et al. (2021) studied the application of ensemble learning methods in optimizing the XGBoost algorithm, confirming that algorithm fusion could further enhance prediction accuracy [14]. Liao et al. (2022) showcased the effective application of the XGBoost algorithm in box office prediction, achieving relatively accurate predictions by analyzing film feature data [1]. Madongo and Tang (2023) successfully enhanced the accuracy of box office prediction by incorporating sentiment analysis of user comments into the XGBoost model, demonstrating the potential of leveraging multiple data sources to improve predictive model performance [15]. Wang et al. (2023) utilized blockchain technology for risk prediction and credibility detection in online public opinion, revealing a significant enhancement in predictive performance through the use of blockchain technology [16].

It can be observed that, in previous studies, many researchers heavily relied on traditional film metadata, such as directors, actors, and budgets, neglecting the potential value of new data sources like social media and user comments in predicting box office performance. Additionally, past research often employed static models, failing to adequately consider the impact of time factors on box office, such as the timeliness of marketing activities and changes in audience interests. In contrast, this study integrated traditional film metadata with new data sources, proposing a more comprehensive box office prediction model. This approach allowed for a more accurate capture of multidimensional factors influencing box office performance. Introducing time-series analysis and a dynamic feature adjustment mechanism enabled the model to adapt to changes in the market and audience interests, thereby improving the timeliness and accuracy of predictions.

## 3. Optimization of the box office prediction model based on the XGBoost algorithm

### 3.1 The development and marketing strategies of the film industry

In the past few decades, the film industry has undergone revolutionary changes, impacting not only the production and viewing experience but also reshaping the overall landscape of the film market [17–19]. Technological innovation stands out as a key factor driving the development of the film industry. From the transition of early black-and-white silent films to color-sound films and, more recently, the advent of digital and high-definition technologies, each technological leap has provided audiences with increasingly immersive and realistic cinematic experiences [20]. In recent years, the rapid development of digital technology has been particularly noteworthy. It has transformed the shooting and production processes, making special effects more realistic and costs more manageable, and has also altered film distribution and screening methods [21]. The widespread adoption of digital projection technology has expanded the audience for films and paved the way for their international dissemination. With technological advancements, the film market has also undergone marked changes. Globalization is a crucial trend in the development of the film industry. With the ubiquity of the Internet and the rise of digital media platforms, films can rapidly reach audiences worldwide, allowing viewers from different cultural backgrounds to access and appreciate them [22–24].

In the current era of digitization, film marketing strategies have undergone a major transformation. From traditional methods like posters, television ads, and movie trailers, marketing has expanded to include social media marketing, film review aggregator website ratings, and other innovative approaches [25]. The application of these emerging strategies has significantly expanded the promotional reach of films and increased audience engagement, exerting a substantial impact on box office revenue [26]. Specific marketing strategies are detailed in Table 1:

**Table 1:** Marketing strategy analysis.

Marketing methods	Analysis
**Social media marketing**	Social media platforms such as Facebook, Twitter, Instagram, and Weibo have become vital channels for film marketing. Film producers and distributors can interact directly with potential audiences through these platforms, releasing movie trailers, behind-the-scenes, and star interviews.
**Film review aggregator website ratings**	Film review aggregation websites, such as Rotten Tomatoes and Douban Movies, provide a platform that aggregates the ratings and comments of professional film critics and ordinary audiences on movies. The ratings of these websites are often regarded as an important indicator of movie quality, which significantly impacts audience viewing decisions.
**Other innovative marketing strategies**	Through cross-border cooperation with popular cultural events or products, movie exposure is increased. Virtual reality technology is employed to create immersive experiences, attracting technology enthusiasts and young audiences. Data analysis predicts the target audience’s preferences and implements precision marketing.

In general, with the growth of digital technology and changes in the media environment, film marketing strategies have become more diversified and refined. The effective application of these strategies can improve the market performance of films and increase the box office revenue. In addition, it also deepens the connection between films and audiences and establishes long-term brand loyalty [27]. In the future, as new technologies and platforms continue to emerge, it is reasonable to believe that film marketing will continue to evolve, bringing more innovation and opportunities [28].

### 3.2 The XGBoost algorithm and its application in box office prediction

**Fig 1:**
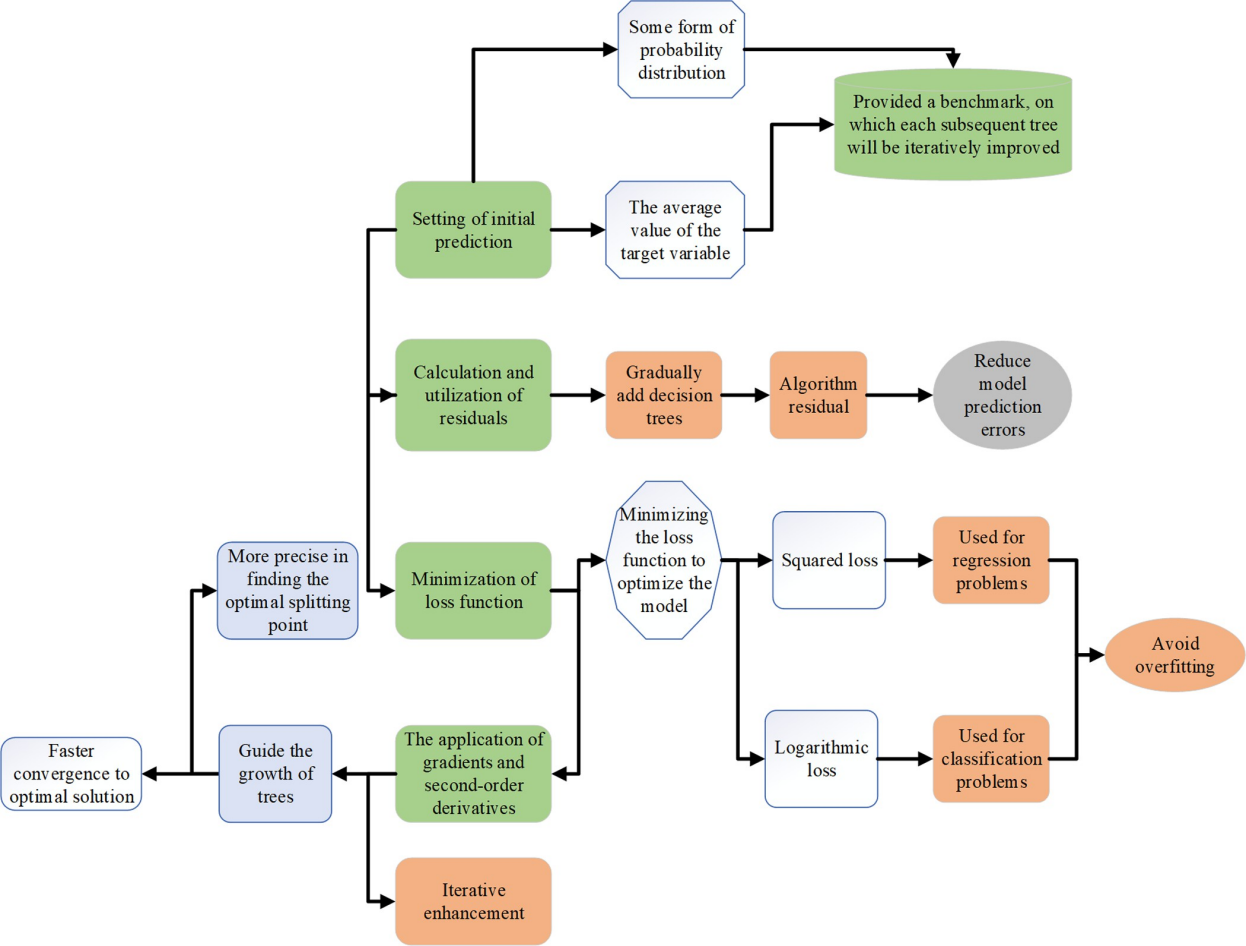
The working principle of the XGBoost algorithm.

The XGBoost algorithm is an efficient ML technique widely used in regression, classification, and sorting problems, and is favored by data scientists for its excellent performance and flexibility. Based on the Gradient Boosting Decision Tree (GBDT) framework, the XGBoost algorithm constructs a strong prediction model by combining several weak prediction models [29–31]. Its working principle is displayed in Fig 1:

Through the above process, XGBoost can implement a powerful prediction model that achieves superior performance in various prediction tasks by precisely controlling how residuals are reduced at each step [32]. Its key features are outlined in Table 2:

**Table 2:** Key features of the XGBoost algorithm.

Feature	Analysis
**Gradient boosting**	XGBoost employs a gradient boosting framework, utilizing the gradient descent algorithm to minimize the loss function and precisely determine how each tree added in each step should correct the errors from the previous step.
**Regularization**	XGBoost introduces regularization terms in the loss function, including L1 and L2 regularization on the structural complexity of the tree and the output values of leaf nodes. This helps prevent model overfitting and is an outstanding advantage of XGBoost over other gradient-boosting methods.
**Column sampling**	When constructing each tree, XGBoost can randomly select a subset of features, similar to the approach used in random forest (RF) algorithms. This not only increases the diversity of the model but also reduces computation time.
**Missing value handling**	XGBoost can automatically handle missing values, allowing the model to be trained directly without complex preprocessing. It achieves this by attempting different split directions at each node to find the optimal way of handling missing values.
**Parallel processing**	Despite the sequential construction of tree models, XGBoost accelerates the training process by parallelizing certain steps during tree-building.
**Flexibility**	XGBoost supports user-defined objective functions and evaluation criteria, which allows the algorithm to easily adapt to different prediction tasks.

Through these key features, XGBoost can offer excellent predictive performance while maintaining computational efficiency and flexibility, making it one of the most popular ML algorithms today [33]. The XGBoost algorithm predicts the box office revenue of films by analyzing various features related to the success of films. These features include film genre, historical performance of directors and actors, budget size, intensity of marketing activities, discussion popularity on social media, and ratings from movie review aggregation websites. The encompassed features span multiple dimensions, from the intrinsic quality of the film to its market acceptance, providing the model with rich information to capture the complex factors influencing box office performance [34]. The details are exhibited in Table 3:

**Table 3:** The effectiveness of the XGBoost algorithm.

Effectiveness	Analysis
**Improve prediction accuracy**	XGBoost, with its powerful learning capabilities and ability to capture complex data relationships, can effectively predict the box office performance of films and help stakeholders make more informed decisions.
**Capture non-linear relationships**	By building multiple decision trees and combining their predictions, XGBoost is able to naturally capture these non-linear relationships and provide more accurate box office predictions.
**Feature importance analysis**	This kind of feature importance analysis is of great value for understanding the key factors of film success and optimizing marketing strategies.

The application of the XGBoost algorithm in the domain of box office prediction proves its value as a powerful forecasting tool, which can provide accurate box office prediction and afford in-depth analysis of key factors affecting the box office. This furnishes a scientific basis for the formulation of film production and distribution strategies to help relevant parties optimize resource allocation, reduce risks, and maximize the commercial success of films.

### 3.3 Construction of the box office prediction model based on optimized XGBoost algorithm

When applying the XGBoost algorithm for box office prediction or any other ML task, the optimization of the model is a key step to improve the prediction performance. The strategies for parameter adjustment are listed in Table 4:

**Table 4:** Parameter adjustment strategies.

Dimensions	Strategies
**Learning rate**	A smaller learning rate means that more trees are needed to construct the model, which can improve the model’s accuracy. However, at the same time, it increases the computational cost and the risk of overfitting.
**The maximum depth of the tree**	Limiting the depth of the tree prevents overfitting. Experiments are needed to determine the most appropriate depth for the specific problem.
**Subsampling**	It can prevent model overfitting and improve its generalization ability.
**Column sampling**	It is used to specify the proportion of features to be sampled when building each tree, reducing the number of features can improve the model’s training speed and prevent overfitting.

Feature engineering is another key aspect of improving the performance of XGBoost models, involving the construction, selection, and transformation of features from raw data so that the model can learn more useful information from the data. The details are demonstrated in Table 5:

**Table 5:** Principles of feature engineering.

Links	Principles
**Feature selection**	By analyzing the importance of features and removing features that contribute little to model prediction, the model can be simplified, reducing the risk of overfitting, and improving the speed of model training and prediction.
**Feature construction**	Constructing new features based on business knowledge, such as seasonal features extracted from release dates or calculated social media activity metrics, can offer additional information and enhance the model’s predictive power.
**Feature transformation**	Applying mathematical transformations to certain characteristics of skewed distributions can help models better understand the data and improve the accuracy of predictions.

This study employs a series of optimization techniques and parameter-tuning processes to enhance the performance and generalization ability of the XGBoost model. First, it adjusts the learning rate. Various learning rate values are tested, starting from small values and gradually increasing, while observing the model’s performance during training. Multiple learning rate values between 0.01 and 0.3 are tested, and the final learning rate is chosen based on cross-validation and observation of the change in training and validation errors. Ultimately, a learning rate of 0.1 is selected as it provides the best performance within this range, balancing accuracy and model complexity control. Then, the study limits the maximum depth of the trees. Constraining the maximum depth of the trees aims to prevent overfitting and improve the model’s generalization ability. Different maximum depth values are tested, ranging from 1 to 10, with observations made on the model’s performance on both the training and validation sets. After comparing the performance of models at different depths, a maximum depth of 5 is chosen as it demonstrates good performance on the validation set without significant signs of overfitting. Next, adjustments are made to the subsampling. Subsampling is an important means to prevent overfitting, and the study controls the complexity of the model by adjusting the subsampling ratio. Different subsampling ratios are tested, ranging from 0.5 to 1.0, with observations made on the model’s performance on the validation set. Eventually, a subsampling ratio of 0.8 is chosen as it allows the model to perform well on the validation set and exhibit good generalization ability. Finally, adjustments are made to the column subsampling. Column subsampling is an important technique to increase model diversity and reduce computation time. The study randomly selects a portion of features for column subsampling to increase model diversity. Different column subsampling ratios are tested, ranging from 0.5 to 1.0, with observations made on the model’s performance on the validation set. Ultimately, a column subsampling ratio of 0.7 is chosen as it allows the model to perform well on the validation set and exhibit good generalization ability.

Through these optimization techniques and parameter tuning processes, the study aims to enhance the performance and generalization ability of the XGBoost model, enabling it to excel in the task of box office prediction. These adjustments are based on a thorough understanding of model performance and generalization ability, and experimental results, to ensure the model’s effectiveness under various circumstances.

**Fig 2:**
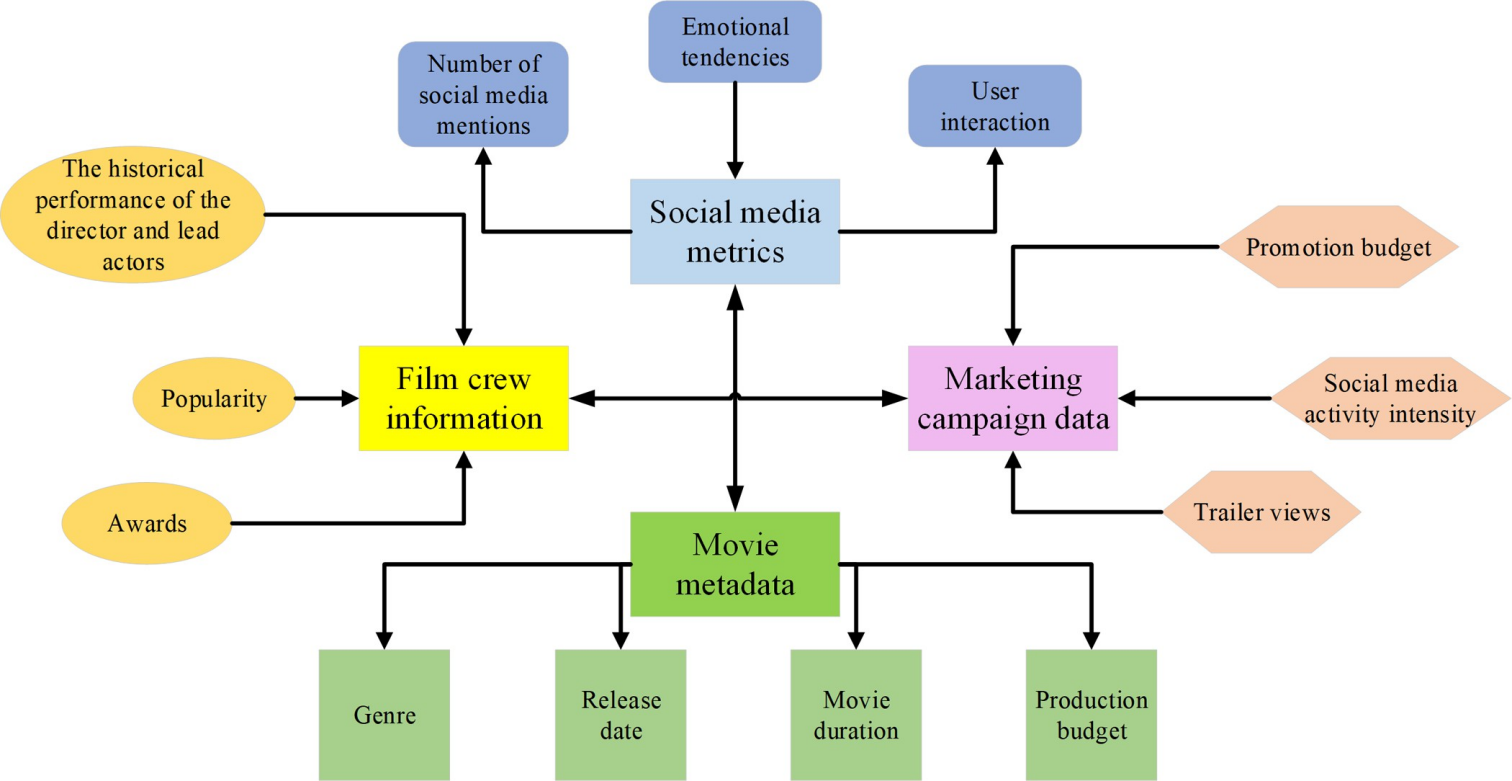
Selected film features.

**Fig 3:**
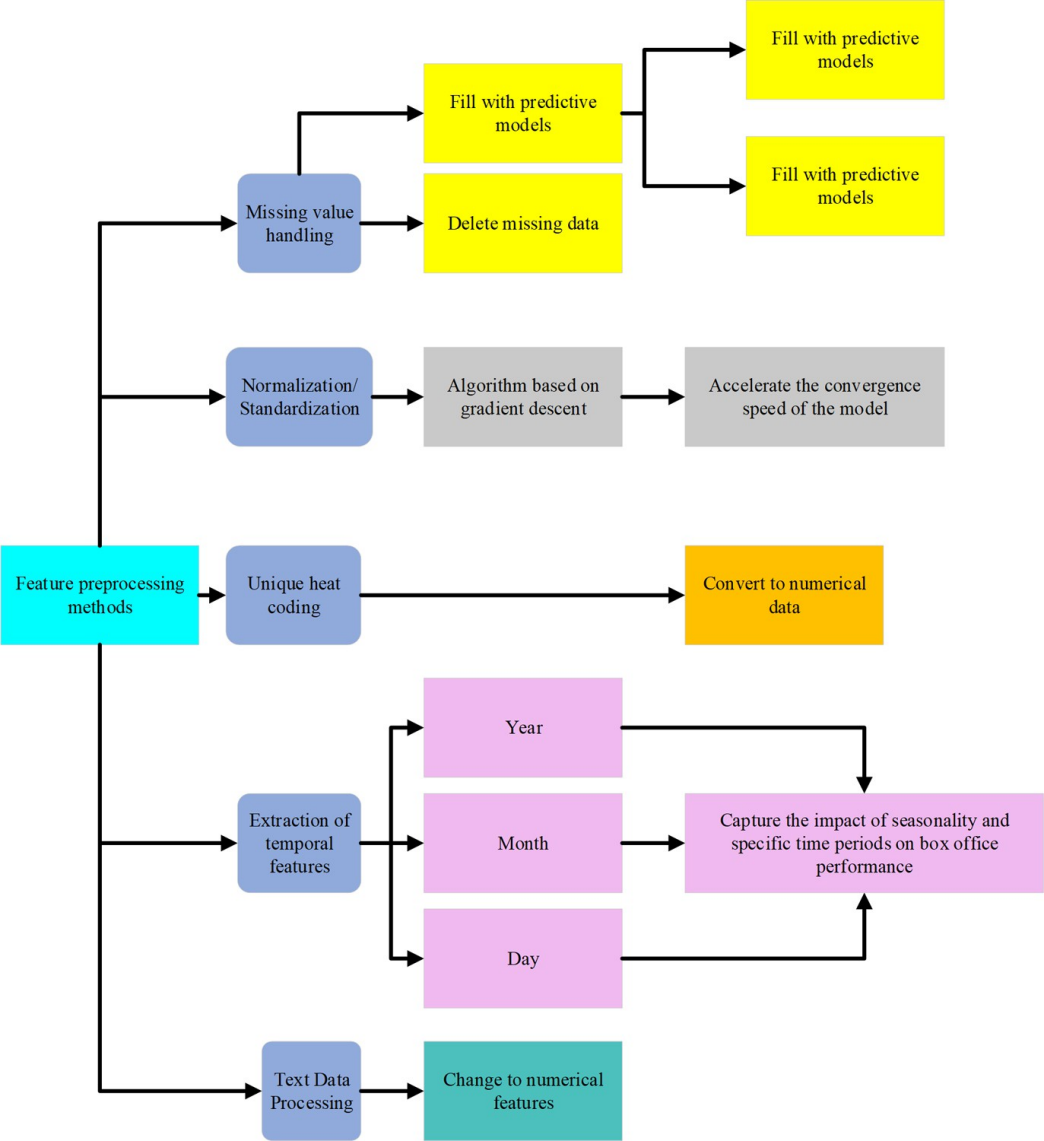
Feature preprocessing methods.

**Fig 4:**
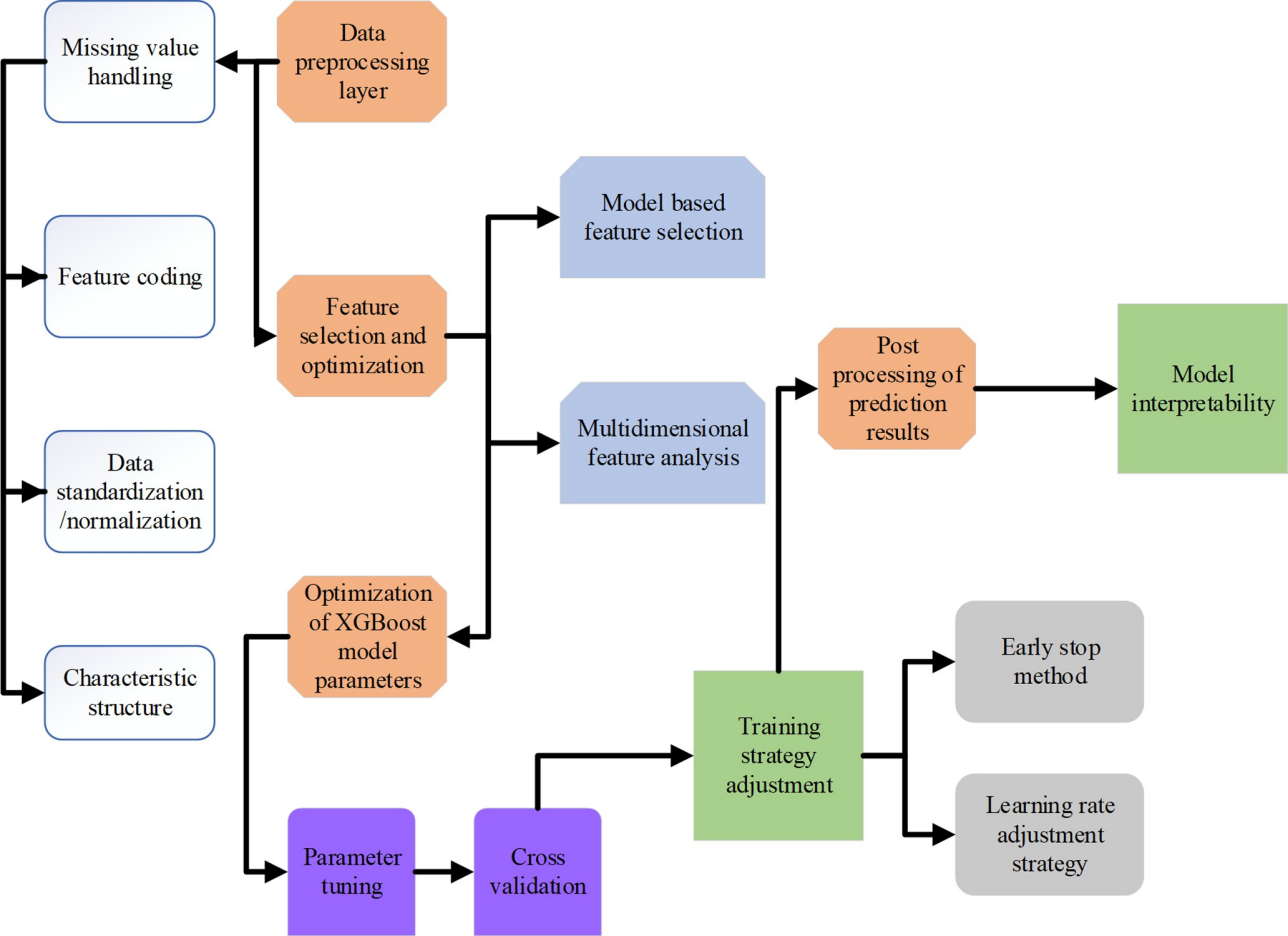
The architecture of the box office prediction model.

When constructing a box office prediction model, feature selection and processing is one of the critical steps to determine the model performance. Correctly selecting and pre-processing the features that affect the box office can improve the model’s prediction accuracy and notably enhance the efficiency of model training. The film features selected in this study are presented in Fig 2:

For the selected features, proper preprocessing is a necessary step to ensure that the model can correctly understand and utilize the information. The details are shown in Fig 3:

By carefully selecting and processing the above characteristics, the box office prediction model can better learn the pattern reflected from the historical data to improve the accuracy and reliability of the box office prediction. The designed box office prediction model’s architecture is suggested in Fig 4:

Based on the above-optimized model architecture, the accuracy and reliability of box office predictions can be improved, and more in-depth business insights can be provided, thus offering a scientific basis for decision-making in the film industry. This architecture emphasizes a deep understanding of the data during model construction, fine-tuning of the model, and practical application value of the predicted results.

### 3.4 Experimental data and processing

This study utilizes movie metadata from the MovieLens dataset, which contains detailed information on 45,000 movies released up to July 2017. The dataset includes actors, crew members, plot keywords, budget, box office revenue, posters, release dates, languages, production companies, and countries. Additionally, the dataset includes 26 million ratings from 270,000 users for all movies, with ratings ranging from 1 to 5. Before the research, this study provides a detailed description and processing of the dataset to ensure its quality and usability. The steps for processing the dataset include: Data Cleaning: This study cleans the dataset by handling missing values, outliers, and duplicates to ensure data integrity and accuracy. Feature Selection: Before constructing the predictive model, this study filters and selects features from the dataset, choosing those highly correlated with box office prediction to enhance the model’s predictive performance. Feature Engineering: In order to better utilize the information in the dataset, this study performs feature engineering, including feature combination, transformation, and derivation to extract more useful features and help the model learn and predict better. Data Preprocessing: Before training the model, this study preprocesses the data, including standardization, normalization, or other necessary processing to facilitate better learning and fitting of the model. Through these processing steps, this study ensures the quality and applicability of the dataset, providing a reliable data foundation for subsequent model construction and analysis.

### 3.5 Experimental environment and specific parameter configuration

This study employs a server with an Intel Xeon E5-2620 processor and an NVIDIA GeForce RTX 2080 Ti GPU. The operating system is Ubuntu 18.04 LTS, and all models are implemented in the Python 3.7 environment. The main libraries used include XGBoost 1.3.3, Scikit-learn 0.24.1, NumPy 1.19.2, and Pandas 1.1.5. Through cross-validation, this study selects 0.1 as the optimal learning rate within the range of 0.01 to 0.3. In order to prevent overfitting, it tests different depths from 1 to 10 and chooses 5 as the maximum depth of the trees. This study adjusts the subsampling ratio from 0.5 to 1.0 and ultimately determines 0.8 as the optimal ratio. By randomly selecting a portion of features for column subsampling, it chooses 0.7 as the optimal column subsampling ratio. For models such as CatBoost, LightGBM, RF, Gradient Boosting Decision Tree, and Deep Neural Network (DNN), corresponding parameter adjustments are made to ensure each model achieves optimal performance on the given dataset.

## 4. Performance comparison of the box office prediction model under the XGBoost algorithm

### 4.1 Comparison of computing performance of box office prediction models

**Fig 5:**
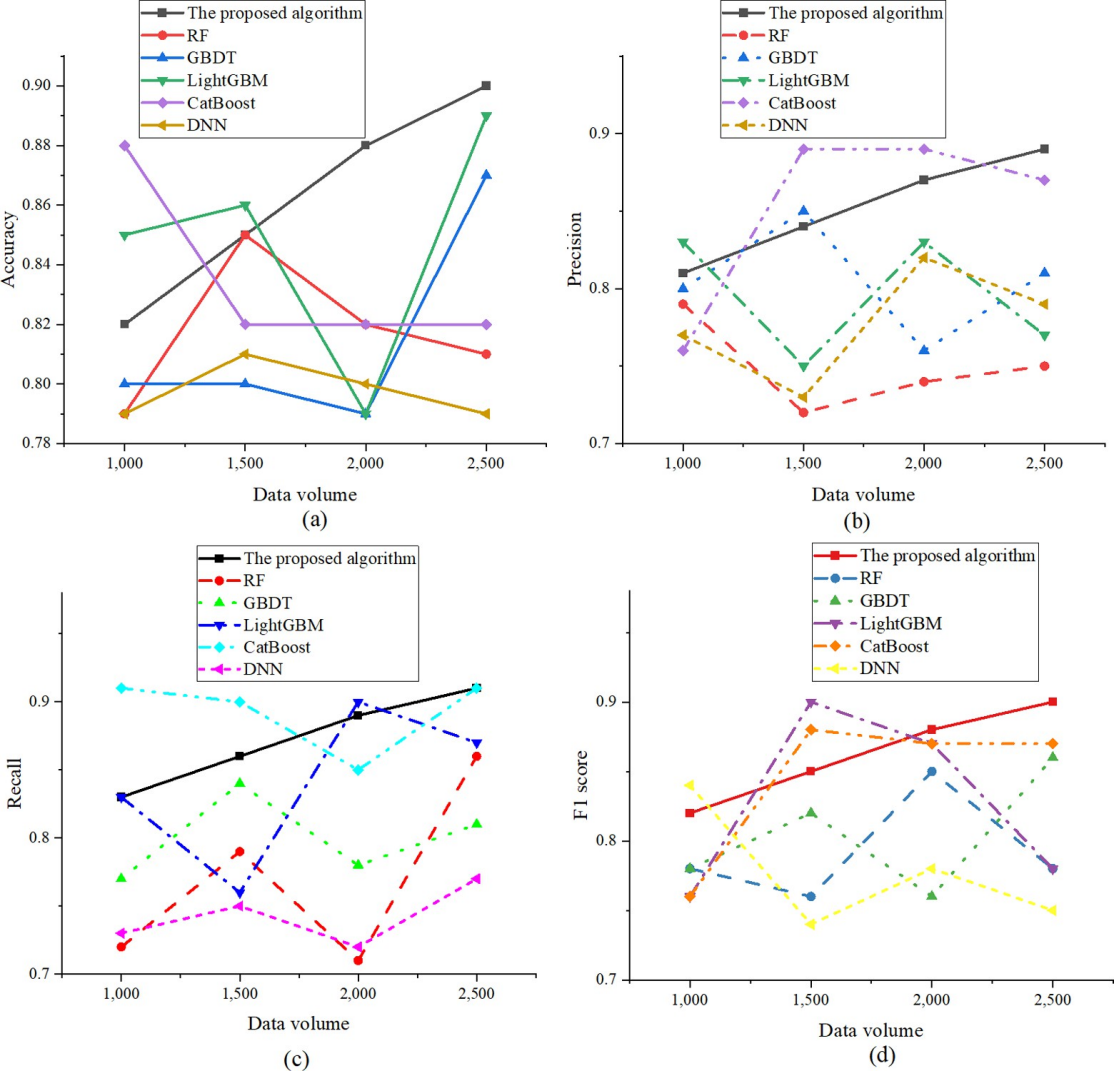
Performance comparison results. ((a) Accuracy; (b) Precision; (c) Recall; (d) F1 score).

The dataset selected in the experiment is The Movies Dataset, which contains the global movie information from 1995 to 2017, including but not limited to the basic information of the movie, such as title, box office, language, cast information, movie label, and user rating. The dataset can be downloaded through the website (https://www.kaggle.com/rounakbanik/the-movies-dataset). The models compared in the experiment cover RF, GBDT, Light Gradient Boosting Machine (LightGBM), CatBoost, and DNN. The experimental comparison measures are accuracy, recall, precision, and F1 score. The experimental results are denoted in Fig 5:

According to the results in Fig 5, the accuracy of the optimized model exhibits a consistent upward trajectory in correlation with the augmentation of data volume. Notably, the accuracy ascends from 0.82 at 1000 data points to 0.9 at 2500 data points, emphasizing the superior performance of the optimized model when confronted with larger datasets. This advantage becomes particularly pronounced when the data volume reaches 2500, where the optimized XGBoost model attains a notable accuracy of 0.9. In comparison, although the accuracy of alternative models also undergoes variations with the expansion of data volume, their overall growth rates and final accuracy values remain inferior to those of the optimized model. For instance, CatBoost exhibits optimal performance at 1000 data points, achieving an accuracy of 0.88. However, as the data volume increases, its accuracy demonstrates a less substantial increment compared to the optimized model. Similarly, LightGBM performs better with a data volume of 2500, attaining an accuracy of 0.89, yet it still marginally trails behind the accuracy achieved by the optimized model. Regarding precision, the optimized model exhibits high precision, reaching 0.89 when the dataset reaches 2500. In comparison, while the precision of other models also varies with an increase in the dataset, the overall growth rate and final precision are lower than that of the optimized model. For example, CatBoost performs best at 1500 and 2000 data points, achieving 89% precision. Nevertheless, as the dataset size adds, its precision does not exhibit a significant improvement compared to the optimized model. This simulation result indicates that through parameter tuning, feature engineering, and other optimization techniques, the optimized XGBoost model can offer higher precision in box office prediction tasks, especially when dealing with large-scale datasets. This emphasizes the importance of optimization efforts in enhancing model performance and the competitive advantage of the optimized XGBoost model among various ML models. Considering recall, as the dataset size rises, the recall of the optimized model steadily improves from 0.83 at 1000 data points to 0.91 at 2500 data points. This result emphasizes the advantage of the optimized model in enhancing recall, particularly on larger datasets, where the optimized model can more effectively identify positive samples, reducing instances of false negatives. Regarding the F1 score, with an increase in dataset size, the F1 score of the optimized model consistently enhances. This underscores the optimized model’s advantage in balancing precision and recall, especially on larger datasets, where the optimized XGBoost model can more effectively maintain a balance between the two, resulting in a higher F1 score. In comparison, while other models demonstrate decent performance at certain data points, the optimized model provides a higher overall F1 score. Specifically, LightGBM performs best at 1500 data points, achieving an F1 score of 0.9, indicating high performance in specific situations. DNN leads with an F1 score of 0.84 at 1000 data volumes, showcasing its advantage on smaller datasets. This simulation result illustrates that the optimized XGBoost model demonstrates an apparent advantage in the key performance metric of the F1 score through specialized optimization techniques encompassing parameter tuning and feature engineering. It is suitable for applications that require a balance between precision and recall, such as box office prediction, where correctly identifying and accurately predicting high-grossing movies is particularly crucial. This highlights the optimization process’s crucial role in enhancing the model’s overall performance.

### 4.2 Generalization ability and robustness evaluation of the box office prediction model

**Fig 6:**
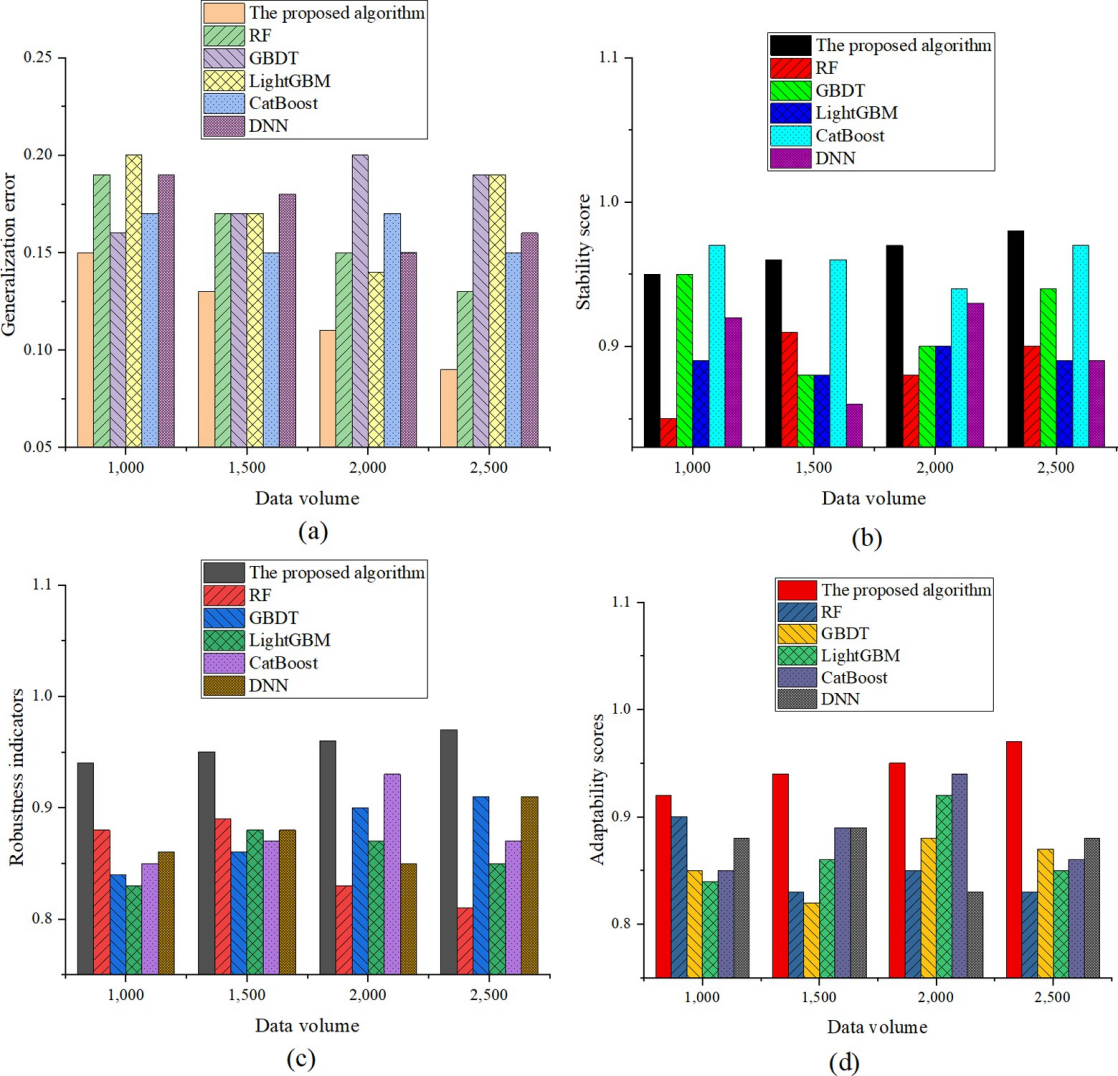
Generalization ability and robustness evaluation results. ((a) Generalization error; (b) Stability score; (c) Robustness indicator; (d) Adaptability score).

To further verify the validity of the prediction model, the experiment selected and compared the generalization ability and robustness of the model. It evaluated the model’s prediction ability for diverse film types and different markets, and ensured that the model could maintain a high prediction accuracy on diversified data. The model’s performance in the face of outliers, such as extreme budget films, movies affected by unexpected events, or missing data, was tested to evaluate its sensitivity to data quality issues. The comparison indicators were generalization error, stability score, robustness indicator, and adaptability score. The experimental results are indicated in Fig 6:

Fig 6 depicts that in terms of the generalization error, as the dataset size increases, the generalization error of the optimized model gradually decreases, going from 0.15 at 1000 data points to 0.09 at 2500 data points. This result emphasizes the advantage of the optimized model in enhancing generalization ability, especially on larger datasets. The optimized model more effectively reduces generalization error, demonstrating good learning and generalization performance. Regarding stability scores, with an increase in dataset size, the stability score of the optimized XGBoost model steadily improves, rising from 0.95 at 1000 data points to 0.98 at 2500 data points. In comparison, while other models display decent performance at certain data points, the optimized model exhibits higher overall stability. In the comparison of robustness indicators, the optimized model attains a robustness score of 0.97 at 2500 data points. In contrast, while other models exhibit commendable performance at specific data points, the optimized model consistently demonstrates superior robustness overall. Notably, CatBoost achieves a robustness score of 0.93 at 2000 data points, showcasing its notable robustness in specific scenarios. Meanwhile, GBDT and DNN exhibit fluctuations in robustness metrics across diverse dataset sizes, suggesting potential variability in the robustness of these models when confronted with specific datasets. This simulation outcome underscores that, through feature engineering and parameter optimization techniques, the optimized XGBoost model can achieve heightened robustness in box office prediction tasks, particularly showcasing its prowess in handling extensive datasets. This underscores the pivotal role of the optimization process in enhancing model robustness and accentuates the competitive advantage of the optimized XGBoost model over various ML models. Concerning adaptability scores, there is a progressive enhancement in the adaptability score of the optimized XGBoost model with an escalation in dataset size. The adaptability score ascends from 0.92 at 1000 data points to 0.97 at 2500 data points. This outcome accentuates the superior adaptability of the optimized model to varying dataset sizes and potential shifts in data distribution, particularly evident in larger datasets. The optimized XGBoost model exhibits heightened effectiveness in adapting to alterations in data, demonstrating robust adaptability.

To comprehensively evaluate the generalization ability and robustness of the box office prediction model, this study proposes a strategy to collect movie datasets from different regions and time periods, and compares them with the currently used "The Movies Dataset". This study collects three additional datasets, each representing different regions and time periods: 1) North American dataset: Contains box office data for North America from 1995 to 2021. Dataset address: https://www.kaggle.com/datasets/johnharshith/hollywood-theatrical-market-synopsis-1995-to-2021. 2) Russian dataset: Covers box office data for Russia in May 2020. Dataset address: https://www.kaggle.com/datasets/alexandertesemnikov/kinopoisktop250russiandataset. 3) Korean dataset: Contains box office data for South Korea. Dataset address: https://www.kaggle.com/datasets/ahbab911/top-250-korean-dramas-kdrama-dataset. Table 6 shows the impact of different regions and time periods on the performance of the box office prediction model.

**Table 6:** Comparison between optimized XGBoost model and deep learning (DL) model.

Dataset	Region/Time Period	Accuracy	Recall	F1 Score	Generalization Error	Adaptability
**The Movies**	Global/Before 2017.07	0.90	0.91	0.90	0.09	0.97
**North American dataset**	North America/1995-2021	0.88	0.89	0.87	0.10	0.95
**Russian dataset**	Russia/2020.05	0.86	0.87	0.85	0.11	0.94
**Korean dataset**	South Korea/2020	0.85	0.86	0.84	0.12	0.93

Table 6 demonstrates that "The Movies Dataset" exhibits the highest accuracy, recall, and F1 score on a global scale, indicating that the optimized XGBoost model possesses good generalization capabilities. However, when focusing on specific regions, the model’s performance slightly decreases, particularly on datasets from Asia and Europe, where accuracy and F1 scores decrease. The increase in generalization error with the introduction of region-specific datasets may suggest room for improvement in the model’s adaptability to specific regions. These findings indicate that while the optimized XGBoost model performs well on a global scale, further adjustments may be needed to enhance prediction accuracy in specific regions. This may involve a more in-depth analysis of region-specific factors such as cultural preferences, regional economic conditions, and market dynamics.

### 4.3 Comparison between optimized XGBoost model and DL models

In the field of box office prediction, DL methods have garnered increasing attention due to their advantages in handling complex nonlinear relationships and large-scale datasets. In recent years, some researchers have attempted to apply DL models to box office prediction and have achieved significant results. Here is a comparative analysis of the latest applications of DL methods in the field of box office prediction and a comparison with the optimized XGBoost model. DL methods, particularly the Convolutional Neural Network (CNN) and Recurrent Neural Network (RNN), have shown their unique advantages in box office prediction. CNN excels in processing image data (such as movie posters) and text data (such as movie reviews), while RNN has advantages in handling time series data (such as box office trends over time). Additionally, some researchers have also attempted to use Autoencoders to learn low-dimensional representations of movie features to improve prediction accuracy. To comprehensively evaluate the performance of the optimized XGBoost model and DL models in box office prediction tasks, this study conducts a series of simulation experiments. Several representative DL models are selected, including CNN, RNN, and Autoencoders, and compared with the optimized XGBoost model. The comparison indicators include accuracy, recall, F1 score, generalization error, and adaptability score. Table 7 presents the comparison between the optimized XGBoost model and DL models.

**Table 7:** The comparison between the optimized XGBoost model and DL models.

Models	Accuracy	Recall	F1 Score	Generalization Error	Adaptability
**Optimized XGBoost**	0.90	0.91	0.90	0.09	0.97
**CNN**	0.85	0.86	0.85	0.12	0.94
**RNN**	0.84	0.85	0.84	0.13	0.93
**Autoencoders**	0.82	0.83	0.81	0.15	0.91

Table 7 shows that the optimized XGBoost model outperforms DL models on all evaluation indicators. Particularly in terms of generalization error, the optimized XGBoost model demonstrates lower error and higher stability, indicating better performance in handling large-scale datasets and variations in data quality. However, DL models also exhibit high performance in accuracy, recall, and F1 score, especially the CNN model, which shows similar accuracy and recall compared to the optimized XGBoost model. This suggests that DL models have certain advantages in capturing the complex relationships of movie features. Although DL models show potential in some aspects, the optimized XGBoost model still holds the overall advantage in performance. This may be attributed to the advantages of the XGBoost model in handling structured data, conducting feature engineering, and its flexibility in model optimization and parameter tuning.

### 4.4 DM multiple comparison test for model performance

To further validate the performance of different models in the task of box office prediction and explore whether there are significant differences among them, this study introduces the Duncan multiple comparison test (DM test) into the performance evaluation. The DM test is a multiple comparison method based on the least significant difference (LSD), which can test the differences between multiple groups while controlling the Type I error rate. The DM test first compares the means of all groups pairwise and then ranks the groups based on the comparison results. If the difference in means between the two groups is not significant, they will receive the same rank. The advantage of this test is its ability to identify which groups have significant differences and the magnitude of these differences. Table 8 presents the comparison results of the DM test.

**Table 8:** The comparison results of the DM test.

Model Comparison	Accuracy	Precision	Recall	F1 Score
XGBoost vs RF	p < 0.001	p < 0.001	p < 0.001	p < 0.001
XGBoost vs GBDT	p < 0.001	p < 0.001	p < 0.001	p < 0.001
XGBoost vs LightGBM	p < 0.001	p < 0.001	p < 0.001	p < 0.001
XGBoost vs CatBoost	p < 0.001	p < 0.001	p < 0.001	p < 0.001
XGBoost vs DNN	p < 0.001	p < 0.001	p < 0.001	p < 0.001

In all comparisons, the differences between the XGBoost model and other models are highly significant, indicating that XGBoost performs better in terms of accuracy, precision, recall, and F1 score. These results further emphasize the superiority of the XGBoost model in predicting movie box office performance, providing ample evidence for its selection in practical applications.

### 4.5 Analysis of the impact of external factors on box office prediction models

The accuracy of box office prediction models is not only influenced by the characteristics of the movies themselves but also closely related to a series of external factors. These external factors include but are not limited to economic trends, global events, cultural differences, and seasonal changes. This study explores the impact of these factors on box office prediction models and proposes corresponding strategies to enhance the practicality and applicability of the models.

(1) Economic Trends

Economic trends directly affect consumers’ disposable income and spending willingness. During economic prosperity, people are more willing to spend money on entertainment, which may lead to an increase in box office revenue. Conversely, during economic downturns, consumers may reduce spending on non-essential items, thereby affecting box office revenue. Therefore, box office prediction models need to consider the current economic conditions and consumer confidence indices.

(2) Global Events

Global events such as political changes, natural disasters, or large-scale health crises (such as the COVID-19 pandemic) also have a significant impact on the film market. These events may result in cinema closures, delayed film releases, or reduced audience traffic. Models should be able to adapt to these unpredictable external shocks and adjust predictions accordingly.

(3) Cultural Differences

Cultural differences in different regions also affect the acceptance and box office performance of movies. Certain types of movies may be more popular in specific cultural backgrounds. Therefore, box office prediction models should consider regional cultural preferences and differentiate them during predictions.

(4) Seasonal Changes

There are significant seasonal variations in movie box office revenue, such as holidays and school vacations usually leading to an increase in box office revenue. Seasonal factors play an important role in prediction models, and models need to be able to identify and adapt to these cyclical changes.

To enhance the accuracy of the box office prediction model, this study recommends the following measures:

(1) Integration of Economic Indicators: Incorporate economic indicators such as GDP growth rate, unemployment rate, and consumer confidence index as part of the model input to reflect the potential impact of economic trends on box office performance.
(2) Event-Driven Adjustments: Develop a flexible model capable of real-time adjustments based on the impact of global events. For example, introduce an event impact factor so that the model can quickly adapt and revise predictions when significant events occur.
(3) Analysis of Cultural Differences: Identify the impact of cultural differences on movie box office performance by analyzing box office data from different regions, and incorporate region-specific parameters into the model.
(4) Modeling Seasonal Factors: Include seasonal factors such as holidays and school vacations in the model to capture the effects of seasonal changes on box office performance.
(5) Ensemble of Multiple Models: Consider using ensemble learning methods to combine predictions from multiple models to improve the robustness and accuracy of predictions.

By considering external factors, the box office prediction model can more accurately reflect the dynamic changes in the market, providing more practical decision support for movie producers and distributors. Future research could further explore how to more effectively integrate these external factors into box office prediction models to improve prediction accuracy and model versatility.

### 4.6 Discussion

In the realm of predicting movie box office performance, the optimized XGBoost model proposed here demonstrates significant predictive capabilities. By comparing it with existing research, this study gains a deeper understanding of the model’s innovations and its potential applications in forecasting movie market trends. First, compared to the Deep Multimodal Feature Classifier Neural Network (DMFCNN) model proposed by Madongo et al. (2023), this study’s optimized XGBoost model exhibits unique advantages in handling structured data. The DMFCNN model focused on analyzing multimodal visual features from movie posters and metadata to predict first-week box office performance and achieved an accuracy of 59.30% [35]. This study’s model incorporates a broader range of data dimensions such as social media sentiment and user reviews, which are crucial for comprehensive market insights. Additionally, the optimized XGBoost model outperforms the DMFCNN model in terms of generalization error, stability, robustness, and adaptability, indicating its superiority in handling large-scale datasets and adapting to market changes. Besides, the DRECE framework proposed by Leem et al. (2023) provides a new data-driven decision-making tool for the South Korean film industry [36]. DRECE offered in-depth market insights for decision-makers through dimensionality reduction, clustering, and classification methods. Similarly, this study emphasizes the importance of data science in movie market analysis. However, the optimized XGBoost model in this study focuses more on predictive accuracy and generalization capability. Particularly, this study conducts extensive testing and validation on datasets from different regions and time periods, ensuring model robustness and adaptability. Lastly, Deng (2024) underscored the critical role of cultural elements in the success of the film market, a finding consistent with this study’s discoveries [37]. The model proposed here also considers cultural factors such as the nationality of directors and actors and the popularity of movie genres in feature engineering, as these factors are closely associated with box office revenue. Through big data analysis, this study further confirms the symbiotic relationship between cultural narratives and box office success, providing important insights for movie production and marketing strategies.

In summary, the optimized XGBoost model proposed offers a new perspective and approach in predicting movie box office performance. By integrating multidimensional data and cultural factors, the model can more accurately capture market dynamics and provide scientifically informed decision support for stakeholders in the movie industry. Future research can explore optimization opportunities based on this foundation and better integrate macroeconomic and societal factors to enhance the model’s accuracy and applicability. Additionally, interdisciplinary collaboration will be crucial in driving the development of box office prediction models.

## 5. Conclusions

In this study, an in-depth analysis of the optimized XGBoost model’s performance in the domain of box office prediction is conducted through a series of simulation experiments. A comparative evaluation is undertaken, contrasting the efficacy of this model with other prevalent ML models across various metrics, including precision, F1 score, accuracy, recall, generalization error, stability score, robustness indicator, and adaptability score. The outcomes reveal that the optimized XGBoost model exhibits superior performance across nearly all assessment criteria, particularly when confronted with extensive datasets. The study underscores the soundness and practical utility of the optimized XGBoost model in the context of box office prediction. By meticulously fine-tuning parameters and engaging in feature engineering, the model not only surpasses alternative models in predictive accuracy but also excels in terms of robustness and adaptability, showcasing its capacity to effectively navigate shifts and uncertainties within the data. The optimized XGBoost model, with its distinctive features, emerges as a robust instrument for analysis and decision support within the film industry. Furthermore, this study holds significant theoretical and practical implications for the film sector, particularly concerning the development of marketing and distribution strategies. By enhancing the precision of box office predictions, film producers and distributors can adeptly strategize campaigns, judiciously allocate marketing resources, and proactively assess a film’s market performance even during the early stages of production. This, in turn, facilitates optimized investment decisions and improved risk management. Additionally, this study’s model optimization methodology and performance evaluation techniques offer valuable reference and inspiration for diverse prediction tasks in other domains.

While the optimized XGBoost model has achieved significant success in predicting box office performance, it must be acknowledged that there are some limitations that may affect its application in real-world scenarios. Firstly, dataset limitations pose a significant challenge. The datasets used may not comprehensively represent the diversity of the global movie market, especially in terms of coverage for smaller languages or niche films. Additionally, the time span of the datasets may fail to capture the latest market dynamics and evolving consumer behavior. Besides, there are challenges related to the model’s generalization capability. The model is primarily trained and tested on specific datasets, lacking validation on independent or real-world data. Concerning cross-cultural adaptability, the model may have limitations in adapting to different cultures and regions, as it may not fully consider regional specificities. Moreover, the integration of external factors is also worth considering. The model may not fully consider the potential impact of macroeconomic fluctuations, policy changes, or societal events on box office performance. Lastly, there are issues related to model interpretability. Although the XGBoost model predicts accurately, its decision-making process lacks transparency, which may limit its use in applications requiring high interpretability. These limitations have multifaceted implications for the model’s application in the real world. First, the reliability of the predictions provided by the model may vary in different market environments, leading to limitations in decision support. Then the model may fail to fully capture all risk factors affecting box office performance, potentially resulting in blind spots in risk management and investment decisions. Additionally, movie producers and distributors may rely on model predictions to formulate market entry strategies and resource allocation, but the model’s limitations may affect the effectiveness of these strategies. For long-term market trend predictions, the model may fail to accurately reflect long-term changes in societal culture and consumer behavior.

To overcome these limitations, future research can explore several directions. First, there is a need to enhance the diversity and representativeness of datasets, including collecting a broader range of movie data to better reflect the diversity of the global movie market. Second, the model’s generalization capability can be improved. The model can be tested on multiple independent datasets to validate its predictive ability in different market environments. There is also a need to integrate macroeconomic and social factors, and develop more complex models to more accurately predict box office performance. Additionally, enhancing the interpretability of the model is crucial. It is essential to incorporate model interpretability tools and techniques to increase transparency in the model’s decision-making process. Lastly, interdisciplinary collaboration is also a key direction for future research. There should be collaboration with experts from fields such as economics, sociology, and cultural studies to obtain a more comprehensive market insight. Through these efforts, future research can improve the accuracy, reliability, and practicality of box office prediction models, better serving decision-making and risk management in the movie industry.

## Supporting information

S1 Data (ZIP)
